# Large lateral tibial slope and lateral-to-medial slope difference are risk factors for poorer clinical outcomes after posterolateral meniscus root tear repair in anterior cruciate ligament reconstruction

**DOI:** 10.1186/s12891-022-05174-3

**Published:** 2022-03-14

**Authors:** Cham Kit Wong, Gene Chi Wai Man, Xin He, Jonathan Patrick Ng, Alex Wing Hung Ng, Michael Tim Yun Ong, Patrick Shu Hang Yung

**Affiliations:** 1 Department of Orthopaedics and Traumatology, Faculty of Medicine, The Chinese University of Hong Kong, Hong Kong, SAR China; 2 Department of Orthopaedics and Traumatology, Prince of Wales Hospital, Hong Kong, SAR China; 3 Department of Imaging & Interventional Radiology, Prince of Wales Hospital, Hong Kong, SAR China

**Keywords:** Meniscus root tear, Anterior cruciate ligament, Lateral tibial slope, Meniscal extrusion, Posterolateral meniscus root tear, Functional outcomes

## Abstract

**Background:**

Meniscus root tear is an uncommon but detrimental injury of the knee. Hoop stress is lost during meniscus root tear, which can lead to excessive tibiofemoral contact pressure and early development of osteoarthritis. Posterolateral meniscus root tears (PLRT) are more commonly associated with anterior cruciate ligament (ACL) tears. As the lateral compartment is less congruent than the medial compartment, it is more susceptible to a shearing force, which is increased in the ACL-deficient knee. In accordance with the compressive axial load, the increase in the tibial slope would generate a greater shearing force. The additional lateral compartment mobility caused by ACL tear should be reduced after ACL reconstruction (ACLR). However, there is a lack of evidence to conclude that ACLR can sufficiently limit the effect of large tibial slope (LTS) on the healing after PLRT repair. This study aimed to evaluate whether a steep LTS would be a risk factor for poorer clinical outcomes after PLRT repair concomitant with ACLR.

**Methods:**

In this retrospective study, a chart review was conducted to identify patients with concomitant unilateral primary ACLR and PLRT repair. Patients with a partial tear or healed tear were excluded. Postoperative MRI and clinical assessments were performed at a mean follow up of 35 months. MRI data was used to measure the LTS, medial tibial slope (MTS), coronal tibial slope (CTS), the lateral-to-medial slope difference (LTS-MTS) and meniscus healing and extrusion. Functional outcomes were evaluated by patient-reported outcomes (International Knee Documentation Committee [IKDC], Lysholm and Tegner scores) and KT-1000 arthrometer assessment. Interobserver reproducibility was assessed by two reviewers.

**Results:**

Twenty-five patients were identified for the analysis. Patients with larger LTS and larger LTS-MTS differences were shown to be correlated with poorer IKDC scores after surgery (R = -0.472, *p* = 0.017 and R = -0.429, *p* = 0.032, respectively). Herein, patients with LTS ≥ 6° or LTS-MTS ≥ 3° demonstrated poorer IKDC scores.

**Conclusion:**

A large LTS (≥ 6°) and a large difference of LTS-MTS (≥ 3°) were shown to be risk factors for poorer functional and radiological outcomes for PLRT repair in patients after ACLR. Clinically, closer monitoring and a more stringent rehabilitation plan for patients with LTS ≥ 6° or LTS-MTS ≥ 3° would be recommended.

## Background

Meniscus root tears are defined as tears that are located within 1 cm of the meniscus insertion or as avulsion of the insertion site [1]. Although less common than meniscal body tears and frequently unrecognized, it can occur in 0.8 to 15% of knee injuries, with a higher incidence associated with an anterior cruciate ligament (ACL) injury [2–4]. Posterolateral meniscus root tears (PLRT) are more common in patients with ACL tears, which occur 10.3 times more likely than posteromedial meniscus root tears (PMRT) [5]. A meniscus root tear is biomechanically comparable to a total meniscectomy as both would lead to compromised hoop stresses. This further results in the decreased tibiofemoral contact area and increased contact pressures in the involved compartment [6, 7], which may eventually lead to the early development of osteoarthritis [8–10]. An intact lateral meniscus is an important secondary stabilizer of ACL-deficient knee under pivot shift loading, whereas PLRT can further increase rotational instability to promote the onset and progression of osteoarthritis [11].

Moreover, the lateral compartment was found to be more susceptible to shearing force. As shown in a previous study, the ACL-deficient knee would cause a significant increase in both anterior tibial translation and internal tibial rotation at a low knee flexion angle [12]. The lateral compartment is less congruent than the medial compartment, which can result in a greater degree of anterior tibial translation. In accordance with the compressive axial load, the increase in the tibial slope would generate a greater shearing force. Based on Kolbe et al*’s* finding, it was demonstrated that a steep lateral tibial slope (LTS) and lateral-to-medial slope difference are risk factors for concomitant PLRT in patients with ACL injuries [13]. However, the association of these risk factors with the poorer outcome after PLRT repair concomitant with anterior cruciate ligament reconstruction (ACLR) remains to be elucidated.

Ideally, the stability of the injured knee is expected to be largely restored after ACLR and lateral compartmental mobility caused by ACL tear would be reduced. However, there is no conclusive evidence to support that ACLR is sufficient to limit the effect of large LTS on clinical outcomes after PLRT repair concomitant with ACLR. Herein, this study aims to evaluate whether a large LTS is a risk factor for poorer outcomes after PLRT repair concomitant with ACLR. We hypothesized that patients with a large LTS or higher LTS-MTS would demonstrate poorer clinical and radiological outcomes after PLRT repair concomitant with ACLR.

## Methods

### Patient selection

A retrospective study was designed to evaluate the association of functional outcomes and the sagittal and coronal slopes of the tibial plateau in ACL-injured subjects undergoing concomitant PLRT repair and ACLR.

**Fig. 1:**
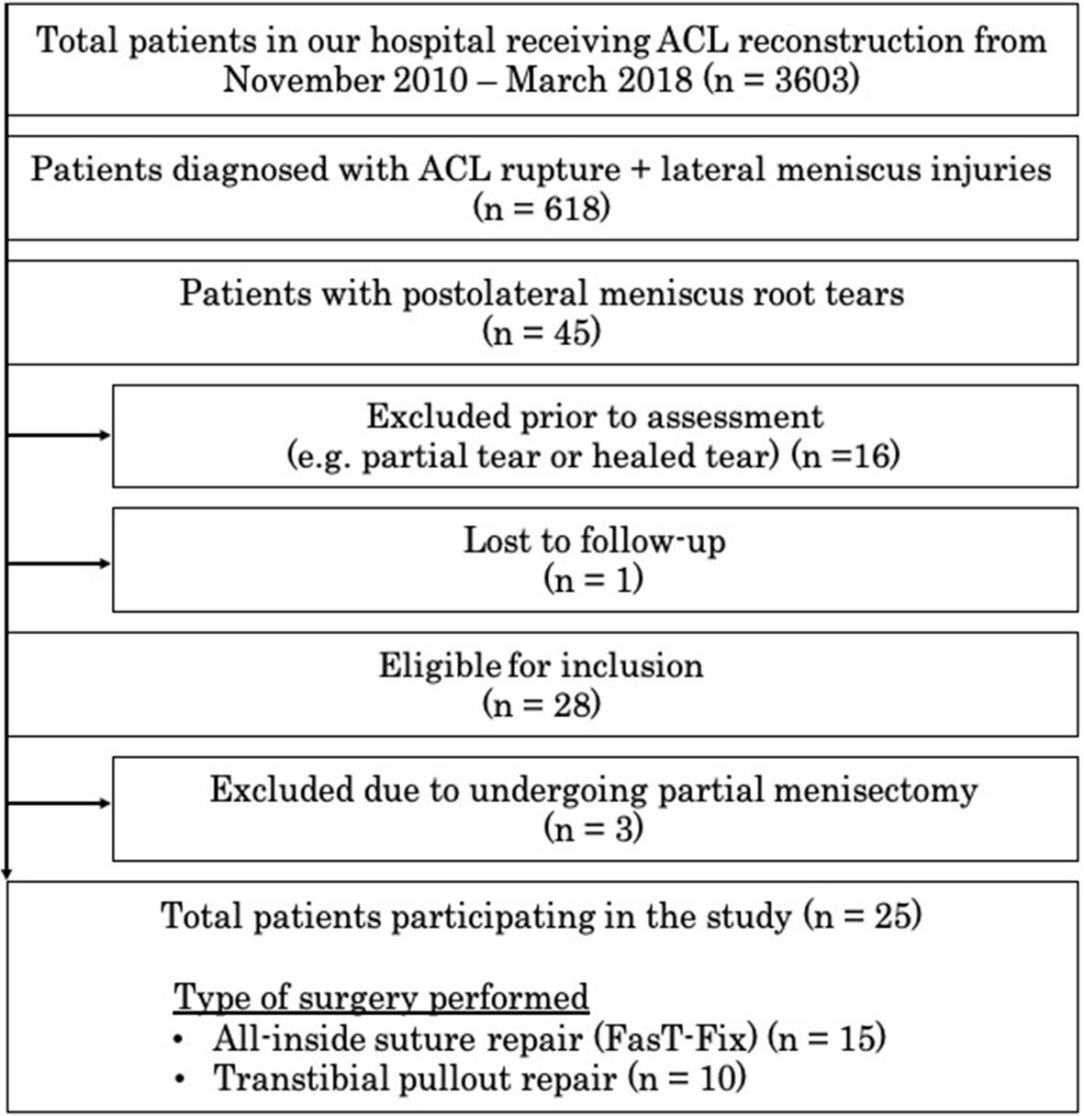
Outline of patients recruited for the current study. ACL, anterior cruciate ligament

The study design was approved by the local ethics committee, and informed consent was obtained from each patient before the start of this study. A chart review was conducted using an electronic medical record system to identify all patients undergoing primary ACLR at the institution between November 2010 and March 2018 (Fig. 1). For this study, only patients aged between 18 to 60 years old with associated PLRT, as confirmed by arthroscopy, were included. Those who had undergone ACLR and PLRT repair were further analysed. PLRT was defined as avulsion injuries of the posterior lateral meniscus root or complete radial tears within 1 cm from the posterior bony insertion of the lateral meniscus [1]. Exclusion criteria included those aged < 18 or > 60 years old and those with genu valgum, pre-existing symptomatic knee osteoarthritis, rheumatoid arthritis, knee range of movement (ROM) < 100°, lateral or medial collateral ligament laxity of grade 3 or higher, a flexion contracture > 10°, lack of available preoperative digital magnetic resonance imaging (MRI) of suitable quality, concomitant of multiple ligament injuries, associated cartilage injury, concomitant tears in other parts of the meniscus other than PLRT, refuses surgical treatment, and a history of previous surgery at the index knee. Patients with partial or healed PLRT were not selected in our study. Patients who defaulted follow-up were also excluded.

### MRI measurement

Imaging was performed on 1.5-T MRI units (GE Healthcare) using dedicated surface multichannel knee coils. The 3D SPGR knee examination was acquired in the sagittal plane. Imaging was performed with a 7.1-ms repetition time, 2.4-ms echo time, 1.4-mm slice thickness, 120-mm field of view, 10^o^ flip angle, 256 × 256 matrix, and 244.14-Hz/pixel bandwidth.

### Slope measurement

All patients’ knee MRIs were reviewed on eUnity platform (Client Outlook Inc.). MRI was first assessed by a board-certified radiologist for image quality assessment. All suitable MRI scans were then used for the determination of the tibial slope. The coronal view was used to measure the coronal tibial slope (CTS), whereas the MTS and LTS were measured on the sagittal view.

**Fig. 2:**
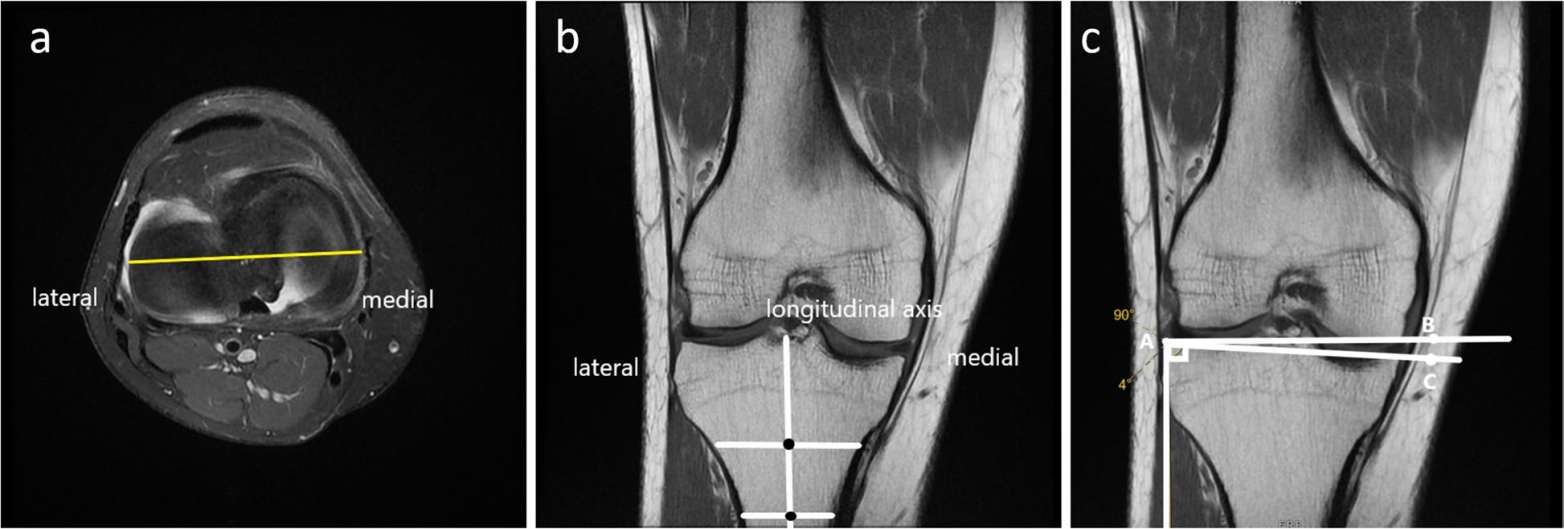
Illustration of coronal tibial slope measurement on T1 MRI sequence. **a** Axial plane through the tibiofemoral joint showing the top view of the tibial plateau. The yellow line represents the coronal plane that passed closest to the centroid of the tibial plateau. **b** Using the coronal view, two lines are drawn across the lateral and medial sides. The coronal longitudinal axis was determined by having a line connecting these two midpoints. **c** The coronal tibial slope was formed as an angle measured by a line drawn along the peak points on the medial and lateral aspects of the plateau and the line perpendicular to the coronal longitudinal (tibial) axis

The CTS was measured on the T1 sequences of the MRI scanning, according to previous methods described [14, 15]. A positive value represents a tibia vara and a negative value represents a tibia valga. In brief, the proximal aspect of the tibial plateau was first identified by placing an axial slice through the tibiofemoral joint (Fig. 2a). Using this section, the coronal plane that passed closest to the centroid of the tibial plateau was then identified. With the coronal view, the longitudinal axis of the tibia was defined. The midpoint of the medial-to-lateral width of the tibia at two points located approximately 4–5 cm apart was then marked. The line connecting these two midpoints would be defined as the coronal longitudinal axis (Fig. 2b). The angle formed between the line drawn along the peak points on the medial and lateral aspects of the plateau and the line perpendicular to the coronal longitudinal axis would be the CTS (Fig. 2c).

**Fig. 3:**
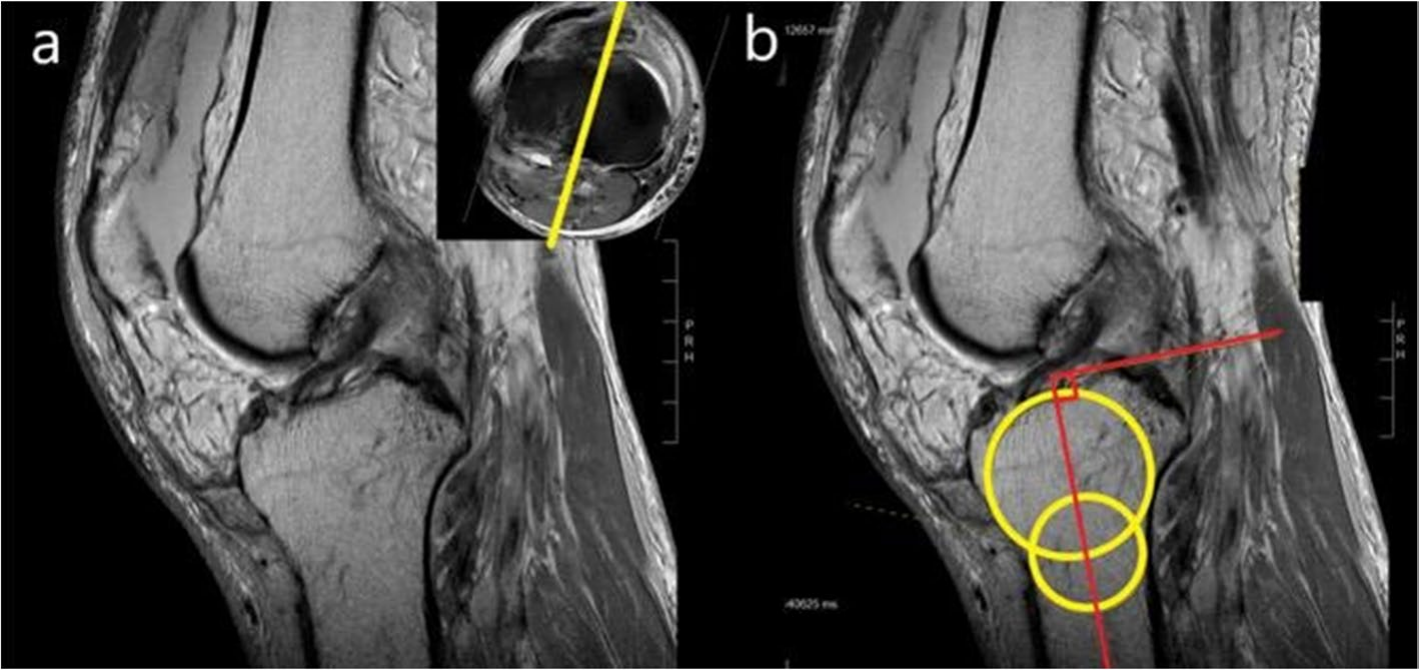
Identification of central sagittal plane on T1 MRI sagittal sequence. **a** The most proximal axial cut of the tibia was identified on MRI (right upper corner). Using this axial cut as scout image, the central sagittal plane was identified. **b** The sagittal longitudinal tibial axis was defined as the line connecting the centres of two circles, then a line is further drawn perpendicular to the longitudinal tibial axis

**Fig. 4:**
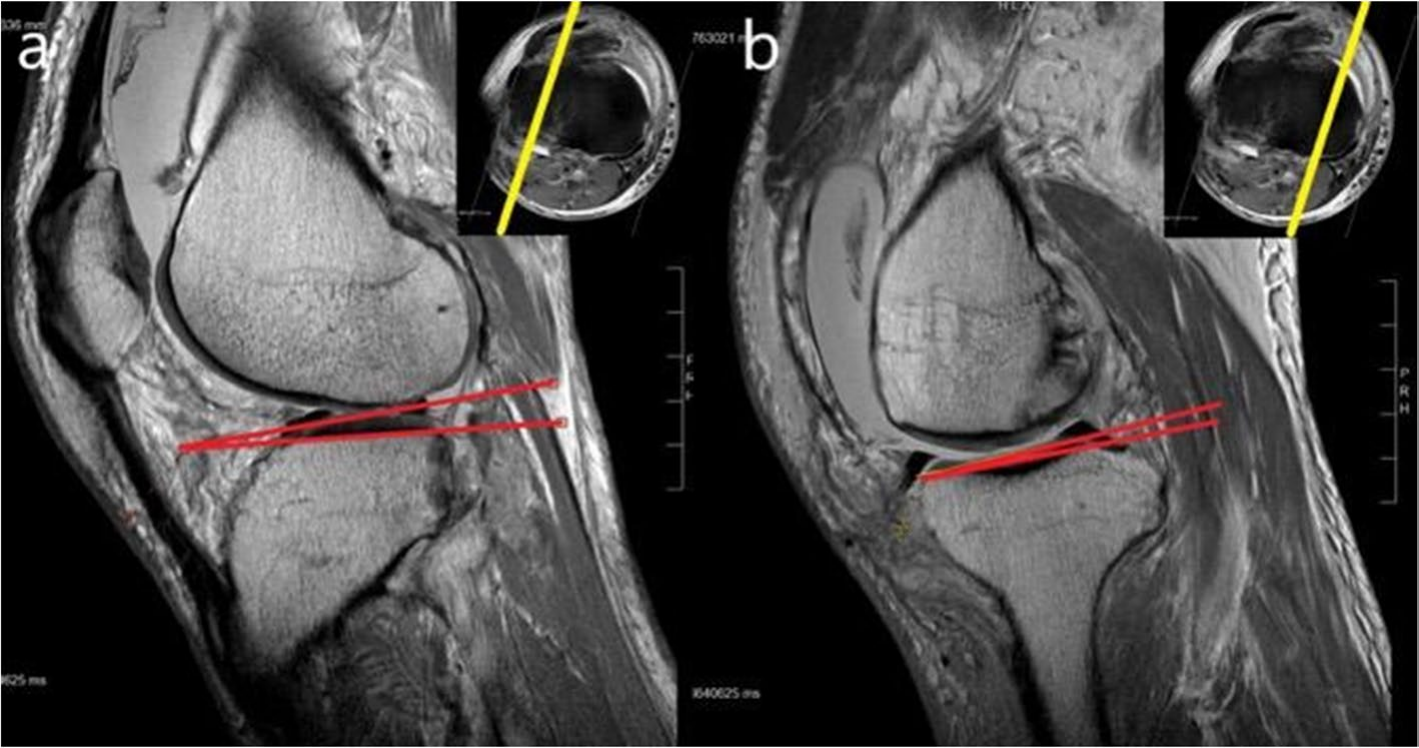
Illustration of sagittal tibial slope measurement on T1 MRI sagittal sequence. The mid-articulating portion of the medial (**a**) and lateral (**b**) plateau were identified and corresponding sagittal images were selected for the measurement of the tibial slope. Using the sagittal images, the plateau slope was drawn connecting peak anterior and posterior points on the plateau. The perpendicular line to the tibial axis was reproduced in this image. The angle between these lines was defined as the tibial slope

“Circle method” was adopted to measure the LTS and MTS [13, 16, 17]. In brief, the most proximal axial cut of the tibia was first identified. The central sagittal plane was identified by using this cut in the scout image (Fig. 3a). Two circles were then drawn in the proximal tibia at this plane. The first circle was drawn touching the anterior, posterior, and proximal cortex. For the second circle, the centre would be positioned at the circumference of the first circle and touching both the anterior and posterior cortex. The sagittal longitudinal tibial axis was defined as a line connecting the centres of these two circles (Fig. 3b). The transverse scout image was used to identify the mid-articulating portion of the medial and lateral plateau. Using the corresponding sagittal images, the plateau slope was drawn connecting peak anterior and posterior points on the plateau. The perpendicular line to the tibial longitudinal axis was reproduced in this image. The angle formed between these lines was defined as the tibial slope (Fig. 4).

In addition, the lateral-to-medial slope (LTS-MTS) difference was assessed as the difference between LTS and MTS. A positive value indicates that LTS was steeper than MTS and a negative value indicates steeper MTS. The MRI examinations were performed at 29.2 ± 25.9 months postoperatively (at least 6 months after surgery). All measurements were performed by two board-certified orthopaedic surgeons on the best agreement basis.

### Determination of meniscal extrusion

Based on the current literature, the lateral meniscal extrusion is defined as significant displacement of the meniscus (≥ 1.1 mm) with respect to the lateral edge of the tibial plateau [18, 19]. As reported in previous literature, the extent of meniscal extrusion was measured in coronal MRIs [20]. At the midpoint of the femoral condyle, two vertical lines were drawn intersecting the margin of the meniscus and the tibial plateau. Osteophytes were excluded for the determination of the margin of the plateau. Displacement of the meniscus from the tibial plateau was measured in millimetres. All post-operative MRIs were assessed by two senior radiologists to determine extrusion.

### Surgical technique and rehabilitation program

All patients had knee arthroscopy to confirm the diagnosis of ACL rupture and PLRT. Knee arthroscopies were performed through standard anterolateral (AL) and anteromedial (AM) portals, distended by the arthroscopic infusion pump. All patients had their surgeries performed at the institutional hospital by two senior orthopaedics specialists. ACLRs were carried out using a hamstring graft with single-bundle technique. During the surgical repair, all PLRT were repaired by either one of the following techniques: all-inside suture repair using FasT-Fix implant [21] or transtibial pullout technique [22]. As previously reported, there was no significant difference between these two types of repair methods toward healing [23]. Thus, the repair methods are not confounding factors to the results of our study.

Toward the post-operative rehabilitation, all subjects underwent the standardized protocol used in our hospital. In short, the meniscal repair was protected by extension knee brace and non-weight-bearing walking with the use of bilateral elbow crutches for 6 weeks. After the protection period, patients underwent the ACL rehabilitation program which was divided into 4 phases. Phase 1 was full range and kinetic chain strength training (week 6–9). Closed chain exercise, gait training, paddle exercise, and balance training were included. This was followed by phase 2 – intensive strengthening and training (week 10–16), with progressive resisted leg press, stepping training and 2D proprioceptive training, and dynamic lunges. The 3rd phase was about functional activity training (week 17–26), in which running and sport-specific training, 3D dynamic proprioceptive training, power training, advanced agility and endurance training and isokinetic resisted program were carried out. In the last phase, patients were directed to return to sports activities gradually (week 26 onwards). Analgesics were prescribed for pain relief. During the rehabilitation period, all patients were closely monitored by a physiotherapist and were regularly followed up by an orthopaedic specialist.

### Clinical assessment

The clinical results were assessed both pre-operatively and two-year post-operatively in all patients. All patients’ data were collected using an academic, web-based documentation platform, comprising three standardized case report forms, completed at the time of surgery and a minimum follow-up of 2 years. The patient-reported outcomes were assessed using standard questionnaires for knee ligament lesions (International Knee Documentation Committee score (IKDC), Lysholm score, and Tegner score). The IKDC score (0–100 point scale) detects improvement or deterioration of knee symptoms, knee function, and sports activities [24]. The Lysholm score (0–100 point scale) detects improvement or deterioration of knee function, particularly symptoms of instability [25]. The Tegner score (0–10 point scale) assesses sport and work activity levels [26]. Bilateral mid-thigh circumference was measured. Quadriceps wasting was defined as mid-thigh circumference on injured side at least 1 cm smaller than the contralateral side. In addition, each patient underwent a KT-1000 arthrometer assessment of anterior tibial translation relative to the femur for laxity of the anterior cruciate ligament by a study-assigned physical therapist post-operatively [27]. The data entry procedure involved several checks of validity and completeness to avoid inappropriate or missing data.

### Evaluation of healing effects

To evaluate meniscal healing, a quantitative estimation of the meniscus was conducted using a 1.5 T MRI with T2 mapping technique preoperatively and postoperatively. The continuity of repaired PLRT on MRIs was documented. Completely healed was defined as the presence of healing over the full length of the tear with a residual cleft < 10% of the thickness of the meniscus. “Incompletely healed” was defined as the presence of healing over the full length of the tear with a residual cleft < 50% of its vertical height. “Not healed” was defined as a residual cleft > 50% of the thickness of the meniscus at any point over the length of the tear [28, 29].

### Statistical analysis

Statistical analysis was performed using SPSS software version 23.0 (IBM-SPSS, New York, USA). Continuous variables were reported as mean ± standard deviation (SD) while categorical variables were described as count and percentages. Pearson / Spearman correlation analyses were performed to evaluate the correlation between demographic data and numeric / nominal outcomes. Two-way mixed intraclass correlation coefficients (ICCs) were used to access the interrater and intrarater reliability and reproducibility. A *p*-value < 0.05 was considered significant for correlations and ICC analysis. ICC < 0.5 is indicative of poor reliability, meanwhile values between 0.5 and 0.75 indicate moderate reliability, values between 0.75 and 0.9 indicate good reliability, and values > 0.90 indicate excellent reliability [30].

## Result

### Patient demographic data

Upon our inclusion and exclusion criteria, 25 patients were eligible for this study. A total of 25 knees were used for the analysis. Among the 25 knees, 10 were left knee and 15 were right knee. The mean age of the study cohort was 29.5 ± 10.5 years. Twenty-one (84%) patients were males and 4 (16%) were females. The demographic characteristics are shown in Table 1.

**Table 1:** Patient demographics (*n* = 25)

Variables	
Gender	
Male	21 (84%)
Female	4 (16%)
Age (years)	29.9 ± 10.5
Time to Surgery (weeks)	42.5 ± 86.7
Time of Operation (minutes)	116.8 ± 43.0
Time from Surgery to Assessments (weeks)	154.2 ± 110.9
Side	
Left	10 (40%)
Right	15 (60%)
Coronal tibial slope (°)	3.3 ± 1.7
Medial tibial slope (°)	4.3 ± 1.2
Lateral tibial slope (°)	6.3 ± 2.6

Data express as mean ± standard deviation, unless otherwise stated

### Reliability of the measurements

The ICC of intrarater reliabilities for MTS, CTS and LTS were 0.996, 0.964 and 0.988 respectively. In addition, the ICC of inter-rater reliabilities for MTS, CTS and LTS were 0.990, 0.959 and 0.986, respectively. Overall, the ICC data suggested excellent measurement consistency for the variables.

### The clinical and radiological outcome with larger lateral and tibial slope

**Fig. 5:**
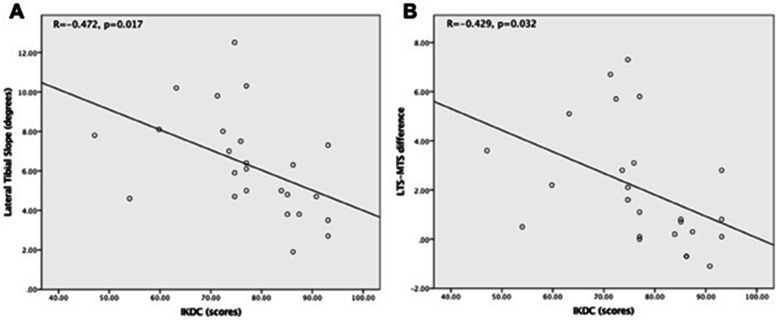
Correlation between lateral tibial slope and IKDC score. **a** Larger lateral tibial slope negatively correlated with IKDC score (R = -0.472; *p* = 0.017). **b** Larger lateral tibial slope to medial slope difference negatively correlates with IKDC score (R = -0.429, *p* = 0.032)

The mean CTS, LTS and MTS were 3.3^o^ ± 1.7^o^, 6.3^o^ ± 2.6^o^ and 4.3^o^ ± 1.2^o^, respectively (Table 1). After receiving the surgical treatment, all subjects noted a general improvement in knee mobility (pre-op Tegner score, 5.8 ± 1.8 vs post-op Tegner score, 7.5 ± 1.5). Postoperatively, patients with larger LTS and larger LTS-MTS differences were negatively correlated with IKDC score (R = -0.472, *p* = 0.017 and R = -0.429, *p* = 0.032, respectively (Fig. 5). In addition, patients with larger LTS-MTS differences had more meniscus extrusion (R = 0.422, *p* = 0.045) documented on MRI. Based on our pivot shift testing, only one patient demonstrated a positive result (grade 1) on the injured knee, whereas the other 24 subjects demonstrated a negative result.

There is no clear definition for an “abnormal” LTS. However, based on the finding from Kolbe R et al. [11], they defined normal LTS as < 6 and abnormal LTS as ≧6. The authors demonstrated a significant difference in occurrence of concomitant PLRT in ACL-injured subjects with LTS <  6^o^ (70%) and ≥ 6^o^ (30%). Comparing our patients with LTS <  6^o^ and ≥ 6^o^, there was no significant difference in the preoperative demographics between the two groups (Table 2). Postoperatively, the patients with LTS ≥ 6^o^ tended to show poorer knee function scores (IKDC score and Lysholm score; Table 3) than those with a LTS <  6^o^ (*p* = 0.053 and *p* = 0.128, respectively). KT-1000 side to side difference (SSD) was not significantly correlated with LTS (R = 0.042, *p* = 0.842). By the KT-1000 correlations, we can conclude that the differences in the outcomes are not related to the knee laxity or ACL repair status.

**Table 2:** Group comparison on patient with difference in lateral tibial slope

Variable	Group (*n* = 25)		*P*-value
	Lateral Tibial Slope		
	< 6 degrees	≥ 6 degrees	
Gender			0.315
Male	11	10	
Female	1	3	
Age (years)	27.8 ± 6.6	31.8 ± 13.0	0.358
Type of Surgery			0.513
Suture	8	7	
Pullout	4	6	
Side			0.870
Left	5	5	
Right	7	8	
Pre-Operative Tegner (scale)	5.8 (2–9)	5.9 (3–10)	0.899
Time of Operation (minutes)	119.8 ± 35.5	113.8 ± 50.2	0.611
Time from Surgery to Assessments (weeks)	153.0 ± 121.7	155.4 ± 105.0	0.959

Data express as mean ± standard deviation, unless otherwise stated

**Table 3:** Patient’s outcome on the difference in lateral tibial slope

Variable	Group (*n* = 25)		*P*-value
	Lateral Tibial Slope		
	< 6 degrees	≥ 6 degrees	
**Patient-reported outcome measure scores postoperatively**			
IKDC Score	82.1 ± 10.9	72.9 ± 11.5	0.053
Lysholm Score	89.8 ± 8.5	83.9 ± 10.3	0.128
Tegner (scale)	7.8 (6–10)	7.1 (5–10)	0.295
**Clinical Outcomes**			
Medial tibial slope (°)	3.8 ± 1.1	4.8 ± 1.2	0.045*
Difference lateral–medial tibial slope (°)	0.5 ± 0.9	3.5 ± 2.5	< 0.001*
Coronal tibial slope (°)	3.8 ± 1.6	2.8 ± 1.8	0.205
Postoperative Extrusion (mm)	1.6 ± 2.0	1.9 ± 1.6	0.738
KT-1000 side-to-side difference, mm	2.3 ± 2.0	2.7 ± 2.5	0.611
< 3 mm	7	8	0.777
3–5 mm	4	3	
> 5 mm	1	2	
Presence of Quadriceps Wasting			0.729
No	6	8	
Yes	5	5	
Postoperative Healing			0.916
Not healed	1	2	
Incompletely healed	1	1	
Completely healed	9	11	

Data express as mean ± standard deviation, unless otherwise stated

*P < 0.05

According to Kolbe R et al. [11], patients with PLRT had a significantly greater difference of LTS–MTS than healthy individuals (3.7 ± 2.9 vs. − 0.6 ± 2.0, respectively). When we stratified the patients into groups of LTS-MTS <  3^o^ and ≥ 3^o^, there was no significant difference in the pre-operative demographics (Table 4). Postoperatively, the group with LTS-MTS ≥ 3^o^ demonstrated a poorer IKDC score (Table 5). When assessing for the power from the IKDC score between LTS-MTS ≥ 3^o^ and LTS-MTS <  3^o^, with the given effect size being 1.23 and at a given alpha 0.05 for a two-sided analysis, the current sample size provided a power of 75.3% in this study.

**Table 4:** Group comparison on patient with difference between lateral tibial slope and medial tibial slope

Variable	Group (*n* = 25)		*P*-value
	Difference of Lateral-Medial Tibial Slope		
	< 3 degrees	≥ 3 degrees	
Gender			0.285
Male	16	5	
Female	2	2	
Age (years)	28.2 ± 6.7	34.1 ± 16.8	0.211
Type of Surgery			0.275
Suture	12	3	
Pullout	6	4	
Side			0.467
Left	8	2	
Right	10	5	
Pre-Operative Tegner (scale)	5.9 (4–10)	5.6 (3–7)	0.705
Time of Operation (minutes)	115.8 ± 36.8	119.0 ± 59.6	0.295
Time from Surgery to Assessments (weeks)	160.1 ± 120.6	139.3 ± 87.4	*0.883*

Data express as mean ± standard deviation, unless otherwise stated

**Table 5:** Patient’s outcome on the difference difference between lateral tibial slope and medial tibial slope

Variable	Group (*n* = 25)		*P*-value
	Difference of Lateral-Medial Tibial Slope		
	< 3 degrees	≥ 3 degrees	
**Patient-reported outcome measure scores postoperatively**			
IKDC Score	80.6 ± 10.9	68.8 ± 10.6	0.022*
Lysholm Score	88.6 ± 9.1	81.9 ± 10.4	0.123
Tegner (scale)	7.6 (6–10)	7.1 (5–10)	0.496
**Clinical Outcomes**			
Postoperative Extrusion, mm	1.6 ± 1.9	2.4 ± 1.4	0.348
KT-1000 side-to-side difference, mm	2.6 ± 2.4	2.2 ± 1.9	0.657
< 3 mm	10	5	0.635
3–5 mm	6	1	
> 5 mm	2	1	
Presence of Quadriceps Wasting			0.759
No	2	1	
Yes	15	5	
Postoperative Healing			0.939
Not healed	10	4	
Incompletely healed	1	0	
Completely healed	7	3	

Data express as mean ± standard deviation, unless otherwise stated

*P < 0.05

## Discussion

The present study illustrated that patients with steeper LTS (≥ 6^o^) and larger LTS-MTS (≥ 3^o^) will have poorer clinical and radiological outcomes after PLRT repair concomitant with ACLR, hence confirming our hypothesis. Although many previous studies have reported the relationship between tibial slope and the incidence of PLRT, the effect of the posterior tibial slope affecting the outcome of PLRT repair after ACLR remains unknown. This is the first study to successfully report the effect of tibial slope on the outcomes after PLRT repair concomitant with ACLR. Importantly, we believe the result from this study can help orthopaedic surgeons to consider whether a closer monitoring and a more conservative rehabilitation plan after PLRT repair would be beneficial to patients with steep LTS and large LTS-MTS difference.

A previous study from Markl et al. reported that larger MTS and LTS were associated with an increased incidence of meniscal lesions when comparing 71 ACL-injured patients with stratification of tibial slope greater or less than 10 degrees [31]. Similarly, Lee et al. compared the incidence of medial meniscal (MM) tears in 174 ACL-injured patients with different LTS (LTS < 13^o^ and LTS ≥ 13^o^) using the lateral view of knee X-rays [32]. It showed the incidence of MM tears was significantly greater for patients with ≥13^o^ LTS (90%) when compared to patients with <13^o^ LTS (58%). In addition, Song et al. compared the LTS of 53 patients with concomitant ACL injuries and medial meniscus ramp lesion with 53 patients with isolated ACL injuries using pre-operative MRI [33]. They found that concomitant ramp lesion was associated with increased MTS.

On the contrary, in a study by El Mansori et al., the authors failed to illustrate the association of increased MTS and the incidence of MM tears [34]. Although the cohort demonstrated that patients with concomitant lateral meniscal tear had increased LTS (9.5^o^) when compared to patients without a meniscal tear (7.2^o^), MTS was not shown to affect the incidence. In Kolbe et al., they measured the tibial slopes of 39 patients with an isolated ACL injury and 20 patients with concomitant PLRT using MRI [13]. As the patients with PLRT were found to have a significantly steeper LTS than control (8^o^ vs 4^o^), they reported the presence of steep LTS and LTS-MTS asymmetry as risk factors for concomitant PLRT in ACL-injured subjects. However, they also failed to find any significant difference in MTS and coronal slope.

KT-1000 side to side differences were shown not correlated with the LTS nor the difference between LTS and MTS. As there was no significant difference among the patient groups, we can assume the ACLR was equally successful in all groups. Herein, we can also speculate that the differences in outcomes are due to the effects of the LTS and LTS-MTS, instead of the ACL status.

Despite the effort of previous studies to correlate tibial slopes and meniscal tear, no evidence of a correlation between tibial slope and outcome after PLRT repair was documented. Hence, this is the first study reporting the impact of LTS on the outcomes of PLRT repair concomitant with ACLR.

Although two different surgical techniques were employed in this study, it has been previously reported that there was no significant difference between the two types of repair [23].

This study may help to provide several clinical implications toward the clinical outcome from PLRT repair with ACLR. Firstly, the prognosis of healing outcomes from ACL-injured patients after PLRT repair is very difficult to predict. Most often this can only be identified when extrusion occurs, and pain develops. Hence, the current study demonstrated that patients with potentially worse outcomes after PLRT repair concomitant with ACLR can be easily identified with LTS ≥ 6^o^ or LTS-MTS ≥ 3^o^. With the early prognosis, stringent conservative postoperative rehabilitation protocol and in-depth monitoring can be adopted during post-operative care for this group of patients. Most importantly, timely management and intervention for these patients can then be provided to cater to individual condition.

There is no consensus on the rehabilitation protocol for PLRT repair. Despite sharing the same basic principles, literature varies regarding ROM restriction guidelines [35, 36]. Many orthopaedic surgeons, including our centre, adopt a single protocol for all meniscus root tears. However, based on the results of our study, a more conservative rehabilitation protocol should be used for patients with a high risk of poorer outcomes (i.e., patients with LTS ≧6 or LTS-MTS > 3). The protected mobilization phase should be prolonged. High-risk patients should be advised to have a longer period of protected weight-bearing, if not non-weight bearing. Hoop stress on the meniscus was created during weight-bearing, and it strains the meniscal roots [37–39]. Micromotion of the repaired meniscus and displacement will occur with cyclic loading [40, 41]. Moreover, active range of motion exercise should be delayed. Hamstring and popliteus are attached to the menisci and their contraction stresses may displace the repair. A longer duration of immobilization should be allowed for the root to heal before allowing active flexion ROM exercise [42].

In this present study, a distinct group of ACL-injured patients undergoing concomitant PLRT repair and ACLR were selected. PLRT with an ACL tear is not a common disease entity. In addition, we have excluded those with a partial tear or healing tear at the time of arthroscopy, which led to the limited sample size. A recent systematic review about “clinical outcomes of surgical repairs for LMPR tears in patients undergoing ACLR” published by Zheng T et al. [43] contained 9 studies with a total of 215 knees only (mean = 19.5, range = 8–41). The present study’s sample size is still larger than the mean of the reported studies.

Despite the aforementioned points, this study has several limitations. Firstly, as this is a retrospective study, the validity of the results may be limited. The present study has a small cohort size (25 patients) owing to the uncommon disease entity of PLRT in an ACL-deficient knee. Some of the statistical significances were marginal or low due to the limited sample size. However, the current sample size can already provide a power of 75.3%. In addition, our study might have neglected other possible factors that could contribute to the outcome differences (e.g. limb alignment, injury mechanism, etc.). Two different operative techniques were used. Despite small scale studies showing no significant difference in outcomes between these two repair methods, large scale high evidence level study is lacking. The difference in repair methods may be a confounder in this study. Moreover, arthroscopic assessment of the meniscus healing is the gold standard and should be adopted for the evaluation of healing status. However, owing to the lack of symptoms, a second look arthroscopy was not favourable to our patients. Thus, postoperative MRI alone was used to assess the meniscus healing. Some previous studies reported outcomes of PLRT repair concomitant with ACLR also used MRI as the sole modality to evaluate post-operative meniscus healing [44–47].

Likewise, further study on how the steeper LTS and larger difference on LTS-MTS can modulate the kinematics and function of the patients’ knees during recovery can help provide a proper rehabilitation strategy. Randomised control trials (RCT) of various rehabilitation plans on high risk (steep LTS and large LTS-MTS) patients can be considered to fill the research gap.

## Conclusion

Our study showed that patients with LTS ≥ 6^o^ and LTS-MTS ≥ 3^o^ would result in poorer knee function after PLRT repair in ACL-injured patients. Importantly, a more conservative rehabilitation plan for patients with LTS ≥6° or LTS-MTS ≥3° should be recommended.

## Data Availability

The datasets generated and/or analysed during the current study are not publicly available due to the privacy and sensitivity of the patients involved but are available from the corresponding author on reasonable request.

## Abbreviations

ACL: Anterior cruciate ligament
ACLR: Anterior cruciate ligament reconstruction
AL: Anterolateral
AM: Anteromedial
CTS: Coronal tibial slope
ICC: Intraclass correlation coefficient
IKDC: International knee documentation committee
LTS: Lateral tibial slope
LTS-MTS: Lateral-to-medial slope
MRI: Magnetic resonance imaging
MTS: Medial tibial slope
NRS: Numerical rating scale
PLRT: Posterolateral meniscus root tears
PMRT: Posteromedial meniscus root tears
ROM: Range of motion
SSD: Side to side difference
SD: Standard deviation
